# U-shaped convolutional transformer GAN with multi-resolution consistency loss for restoring brain functional time-series and dementia diagnosis

**DOI:** 10.3389/fncom.2024.1387004

**Published:** 2024-04-17

**Authors:** Qiankun Zuo, Ruiheng Li, Binghua Shi, Jin Hong, Yanfei Zhu, Xuhang Chen, Yixian Wu, Jia Guo

**Affiliations:** ^1^Hubei Key Laboratory of Digital Finance Innovation, Hubei University of Economics, Wuhan, Hubei, China; ^2^School of Information Engineering, Hubei University of Economics, Wuhan, Hubei, China; ^3^Hubei Internet Finance Information Engineering Technology Research Center, Hubei University of Economics, Wuhan, Hubei, China; ^4^Medical Research Institute, Guangdong Provincial People's Hospital (Guangdong Academy of Medical Sciences), Southern Medical University, Guangzhou, China; ^5^School of Foreign Languages, Sun Yat-sen University, Guangzhou, China; ^6^Faculty of Science and Technology, University of Macau, Taipa, Macao SAR, China; ^7^School of Mechanical Engineering, Beijing Institute of Petrochemical Technology, Beijing, China

**Keywords:** hierarchical topological transformer, multi-level temporal-correlated attention, central connectivity perception, time-series restoration, multi-head attention, brain neurological disease

## Abstract

**Introduction:**

The blood oxygen level-dependent (BOLD) signal derived from functional neuroimaging is commonly used in brain network analysis and dementia diagnosis. Missing the BOLD signal may lead to bad performance and misinterpretation of findings when analyzing neurological disease. Few studies have focused on the restoration of brain functional time-series data.

**Methods:**

In this paper, a novel *U*-shaped convolutional transformer GAN (UCT-GAN) model is proposed to restore the missing brain functional time-series data. The proposed model leverages the power of generative adversarial networks (GANs) while incorporating a *U*-shaped architecture to effectively capture hierarchical features in the restoration process. Besides, the multi-level temporal-correlated attention and the convolutional sampling in the transformer-based generator are devised to capture the global and local temporal features for the missing time series and associate their long-range relationship with the other brain regions. Furthermore, by introducing multi-resolution consistency loss, the proposed model can promote the learning of diverse temporal patterns and maintain consistency across different temporal resolutions, thus effectively restoring complex brain functional dynamics.

**Results:**

We theoretically tested our model on the public Alzheimer's Disease Neuroimaging Initiative (ADNI) dataset, and our experiments demonstrate that the proposed model outperforms existing methods in terms of both quantitative metrics and qualitative assessments. The model's ability to preserve the underlying topological structure of the brain functional networks during restoration is a particularly notable achievement.

**Conclusion:**

Overall, the proposed model offers a promising solution for restoring brain functional time-series and contributes to the advancement of neuroscience research by providing enhanced tools for disease analysis and interpretation.

## 1 Introduction

The blood oxygen level-dependent (BOLD) signal derived from functional neuroimaging is commonly used in brain disorder analysis. As a common brain disorder, Alzheimer's disease (AD) is a progressive neurodegenerative condition characterized by cognitive decline, memory impairment, and changes in behavior (Knopman et al., 2021). The exact cause of AD is not fully understood, but it involves the accumulation of abnormal proteins in the brain, particularly beta-amyloid plaques and tau tangles. To treat brain disorders (e.g., AD, Parkinson's disease), deep brain stimulation (DBS) is a possible way to solve the problem of movement disorders (Limousin and Foltynie, 2019; Ŕıos et al., 2022). It is a neurosurgical procedure that involves the implantation of electrodes into specific regions of the brain to modulate its electrical activity (Leoutsakos et al., 2018). DBS has been investigated as a potential treatment for AD because it offers a way to modulate brain activity in specific areas that are associated with memory and cognition. The electrodes play a critical role in the DBS procedure. These thin, insulated wires are surgically implanted into the brain region of interest. Once in place, they are connected to an implanted pulse generator, which delivers electrical impulses to the brain. These electrical pulses can help regulate the abnormal brain activity associated with certain neurological disorders, potentially improving symptoms (Medtronic, 2020; Alajangi et al., 2022). When it comes to AD, researchers can explore the use of DBS to target brain regions such as the fornix, which is involved in memory and learning. By stimulating these areas, the DBS is able to help improve cognitive function in cognitive patients (Neumann et al., 2023; Siddiqi et al., 2023; Vogel et al., 2023).

**Figure 1:**
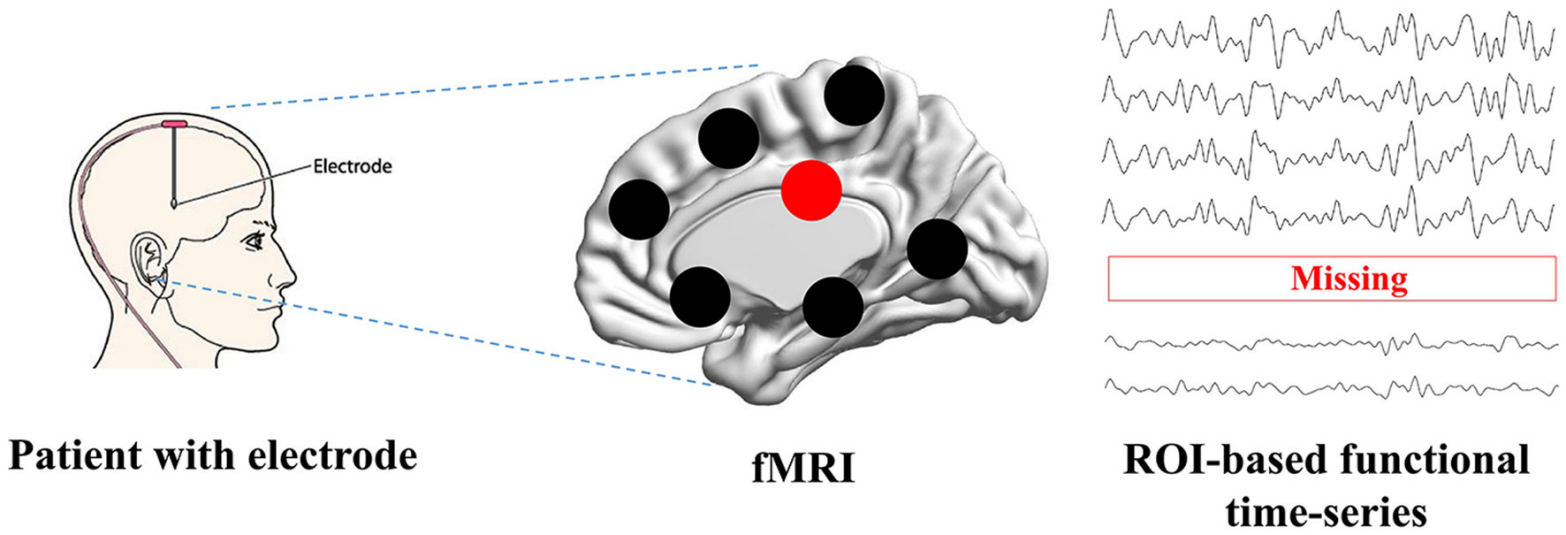
The problem definition. Patients with brain disorders are inserted with the electrodes, which can cause the signal loss when scanning function MRI.

Functional Magnetic Resonance Imaging (fMRI) has revolutionized the field of neuroscience, particularly in the study of brain diseases such as Alzheimer's disease (Forouzannezhad et al., 2019; Yin et al., 2022; Zuo et al., 2023). fMRI is a non-invasive neuroimaging technique that provides valuable insights into the functioning of the human brain by measuring blood oxygenation level-dependent (BOLD) signals. fMRI has been confirmed as a reliable instrument to investigate the brain's functional aspects and explore the brain's mechanisms, enabling early detection, understanding cognitive disease progression, and assessing the impact of interventions (Warren and Moustafa, 2023; Yen et al., 2023). Many studies (Wang et al., 2018; Ibrahim et al., 2021; Sendi et al., 2023) have constructed connectivity-based features and analyzed cognitive disease from fMRI. The constructed features in non-Euclidean space can establish relations between distant brain regions, which is superior than the image-based features in Euclidean space (Chen et al., 2023; Wan et al., 2023d). When evaluating the treatment's performance, fMRI allows researchers to monitor and assess changes in brain activity before and after DBS treatment (Boutet et al., 2021; Soleimani et al., 2023). However, fMRI can sometimes be impacted by the presence of implanted electrodes. The metallic components of these electrodes can create artifacts in the MRI images, which may lead to signal loss or distortion in the region of interest (ROI) (Nimbalkar et al., 2019; Luo et al., 2022; Wang X. et al., 2022). These artifacts include signal intensity changes and temporal and spatial variability. Presently, there are no post-processing MRI techniques available to effectively mitigate these artifacts. Therefore, identifying these specific characteristics is essential for restoring neural activity from artifacts and ensuring the accuracy and validity of fMRI findings in clinical and research settings. Researchers need to address issues related to side effects and fMRI signal loss to further our understanding of the technique's effectiveness in treating this complex and devastating neurodegenerative disease. The possible way to solve this issue is to construct a deep learning model to recover missing signals, as it has achieved complex tasks in medical image analysis (Wang S. et al., 2022; You et al., 2022; Hu et al., 2023; Wan et al., 2023e). As shown in Figure 1, when patients are treated by electrode stimulation, the brain fMRI suffers from signal loss in the stimulated brain regions.

Generative adversarial networks (GANs) have gained prominence in the fields of medical image analysis (Hong et al., 2022) and functional time series reconstruction as a powerful tool for generating synthetic data that closely resemble real-world time series data (Luo et al., 2018, 2019). In addition, Transformer's self-attention mechanism has been successfully applied in medical data analysis (Li et al., 2023; Wan et al., 2023a,b,c). The parallel processing capability and adaptability to various data types make it a versatile tool for time series generation (Tang and Matteson, 2021; Zerveas et al., 2021). Therefore, combining the GAN and transformer can enable the reconstruction of missing time series. Transformer GANs (Generative Adversarial Networks with a Transformer architecture) have been applied to time series reconstruction, offering innovative solutions to various data reconstruction tasks (Wu et al., 2020; Li et al., 2021; Li X. et al., 2022). In many domains, time series data may have missing or incomplete observations. Transformer GANs can be trained to impute the missing data by learning the underlying patterns and relationships in the time series. The generator network creates synthetic data points to fill in the gaps, while the discriminator evaluates the realism of the imputed values. Transformer GANs offer advantages for time series reconstruction due to their ability to capture long-range dependencies and complex patterns and their interpretability through attention mechanisms (Jiang et al., 2021; Zhao et al., 2021). However, these models cannot capture topological relationships at different temporal resolutions, which may degrade the reconstruction performance and the ability to analyze the brain network.

The DBS has emerged as a promising therapeutic approach for Alzheimer's disease (AD), offering potential benefits in alleviating symptoms and modifying disease progression. Although still in the investigational stage, DBS for AD holds promise as a novel intervention aimed at improving cognitive function and quality of life for individuals affected by this devastating neurodegenerative disorder. Among the publicly available datasets, the representative dataset containing brain imaging data for all stages of AD is the Alzheimer's Disease Neuroimaging Initiative (ADNI). Currently, there are no patients implanted with intracortical electrodes for DBS treatment. Our study is the first to theoretically remove some ROI's signals and then utilize our model to recover the removed signals. In this study, we propose a novel U-shaped convolutional transformer GAN (UCT-GAN) model to restore the missing brain functional time-series. First, the fMRI is preprocessed to obtain the ROI-based functional time series. Then, we exclude some ROIs' time-series and treat them as a missing signal. The rest of the ROI-based time-series are sent to the U-shaped topological transformer generator to recover the missing time-series by capturing complex temporal patterns and relationships. Next, the recovered time-series from the generator is sent to the discriminator, consisting of multi-head attention and central connectivity perception, to evaluate the realism of the generated data compared with real fMRI data at different scales. Both spatiotemporal and connectivity features are utilized to constrain the generated missing signals. Finally, we implement a loss function that enforces consistency across different temporal resolutions. This loss encourages the generator to capture diverse temporal scales in the data. When the training reaches Nash equilibrium, the model can recover the missing time-series signal. The main works of this study are as follows:

- The proposed model leverages the power of generative adversarial networks (GANs) while incorporating a U-shaped architecture to effectively capture both global and local features in the restoration process. The temporal characteristics of missing time series can be highly recovered for downstream brain network analysis.
- The multi-level temporal-correlated attention in the transformer-based generator is devised to model the temporal relationship between the missing ROI and other normal ROIs. The topological properties of the missing time-series can be well explored.
- By introducing multi-resolution consistency loss, the proposed model can promote the learning of diverse temporal patterns and maintain consistency across different temporal resolutions, thus effectively restoring complex brain functional dynamics.

## 2 Related work

Reconstructing time-series data using generative adversarial networks (GANs) is a burgeoning field of research with several related studies. GANs offer the potential to generate synthetic time-series by capturing statistical and temporal characteristics. The main advantage of GANs is that they can be used to augment existing time-series datasets by generating additional synthetic data. Increasing the data size is particularly valuable when training machine learning models in medical image analysis. Considering the architecture of the generator, we divide the GAN-based models into two groups: recurrent neural network (RNN)-based approaches and transformer-based approaches.

Mogren (2016) combined RNN and GAN to synthesize more realistic continuous sequential from random noise. Similarly, Esteban et al. (2017) embedded the RNN into both the generator and discriminator to synthesize realistic medical time-series signals by introducing label constraints. Meanwhile, Donahue et al. (2018) introduced the WaveGAN model to generate time-series waveforms by applying one-dimensional convolution kernels. To preserve temporal dynamics, Yoon et al. (2019) proposed the TimeGAN framework to project temporal features onto embedded space through supervisory and antagonistic learning and generate realistic time-series signals to preserve temporal correlation between different variables. In addition, Ni et al. (2020) proposed the SigCWGAN to capture the temporal dependencies inherent in joint probability distributions within time-series signals. Nonetheless, RNN-based approaches are challenging for generating long synthetic sequences. This stems from the sequential processing of time steps in time-series data, where recent time steps exert a stronger influence on the generation of subsequent time steps. Therefore, RNNs fail to establish relationships between distant time steps in lengthy sequences.

Reconstructing time-series signals using transformer GANs, which combine the transformer architecture with GAN, has the potential to capture complex temporal relations of long sequencial time-series. Li X. et al. (2022) successfully designed the generator and discriminator with transformer to synthesize long-sequence time-series signals. Srinivasan and Knottenbelt (2022) proposed the TST-GAN to solve the problem of errors accumulating over time when synthesizing temporal features. This model can accurately simulate the joint distribution of the entire time-series, and the generated time-series can be used instead of real data. To synthesize multivariate time-series within the overall distribution, Madane et al. (2022) introduced different conditions into the transformer-based GAN to approximate the joint distribution of multiple time-series. Li Y. et al. (2022) pointed out the weakness of short-term dependencies in transformers and proposed adversarial convolutional transformers (ACTs) to pay attention to local information of time series. They greatly improved the forecasting accuracy of time-series datasets. Xia et al. (2023) also combined the convolutional networks and transformer in the adversarial training to preserve both global and local temporal features in the time-series generation. However, these models fail to capture the hierarchical temporal features and ignore the temporal characteristics of different frequencies, which may hinder synthesis performance during the time-series generation.

Considering the shortcomings of related methods, we incorporated transformer-based networks into a U-shaped architecture to model temporal relationships on both global and local scales. In addition, the restoration process of time-series is to learn complex distribution, where the generative adversarial networks (GANs) show great ability in learning the underlying patterns and relationships in the time series. Therefore, we try to combine the U-shaped convolutional transformer and GANs to restore the missing brain functional time series for dementia diagnosis.

## 3 Materials and methods

### 3.1 Data description

The Alzheimer's Disease Neuroimaging Initiative (ADNI) dataset^1^ is a comprehensive and widely used resource for studying Alzheimer's disease and related neurological conditions. It includes a variety of data types, including structural and functional MRI (fMRI) data. In this study, we successfully downloaded about 311 subjects from the ADNI website. The patients scanned with fMRI are distributed among the normal controls (NC), early mild cognitive impairment (EMCI), and late mild cognitive impairment (LMCI). The numbers for the three categories are 105, 110, and 96, respectively. The time of repetition (TR) is 3.0 s. The scanning time for each subject is ~10 min.

Preprocessing fMRI data typically involve several steps to ensure data quality and prepare it for analysis. We use the routine GRETNA (Wang et al., 2015) software to preprocess the fMRI to construct multi-ROI time-series. The general preprocessing steps (Zuo et al., 2022, 2024) are as follows: convert the DICOM files into NIfTI format for easier handling and compatibility, remove the first 10 volumes, correct for differences in acquisition times between slices to ensure temporal alignment, correct for head motion during scanning, register the fMRI data to a standard anatomical template (e.g., MNI152) to ensure spatial consistency across subjects, apply spatial smoothing to the data to improve the signal-to-noise ratio and compensate for small anatomical differences between subjects, apply temporal filters to remove low-frequency drifts (e.g., high-pass filtering) and to attenuate high-frequency noise (e.g., Gaussian or bandpass filtering), register the fMRI data to the structural MRI data for each subject, and wrap the fMRI volumes into the automated anatomical labeling (AAL) atlas (Tzourio-Mazoyer et al., 2002) to obtain the time-series of 90 ROIs. At last, the output is the multi-ROI time-series **S**_*e*_ with the size *N*×187. In the following experiments, we remove one or more ROI time-series from **S**_*e*_ and recover the removed time-series through the proposed model.

### 3.2 Architecture

**Figure 2:**
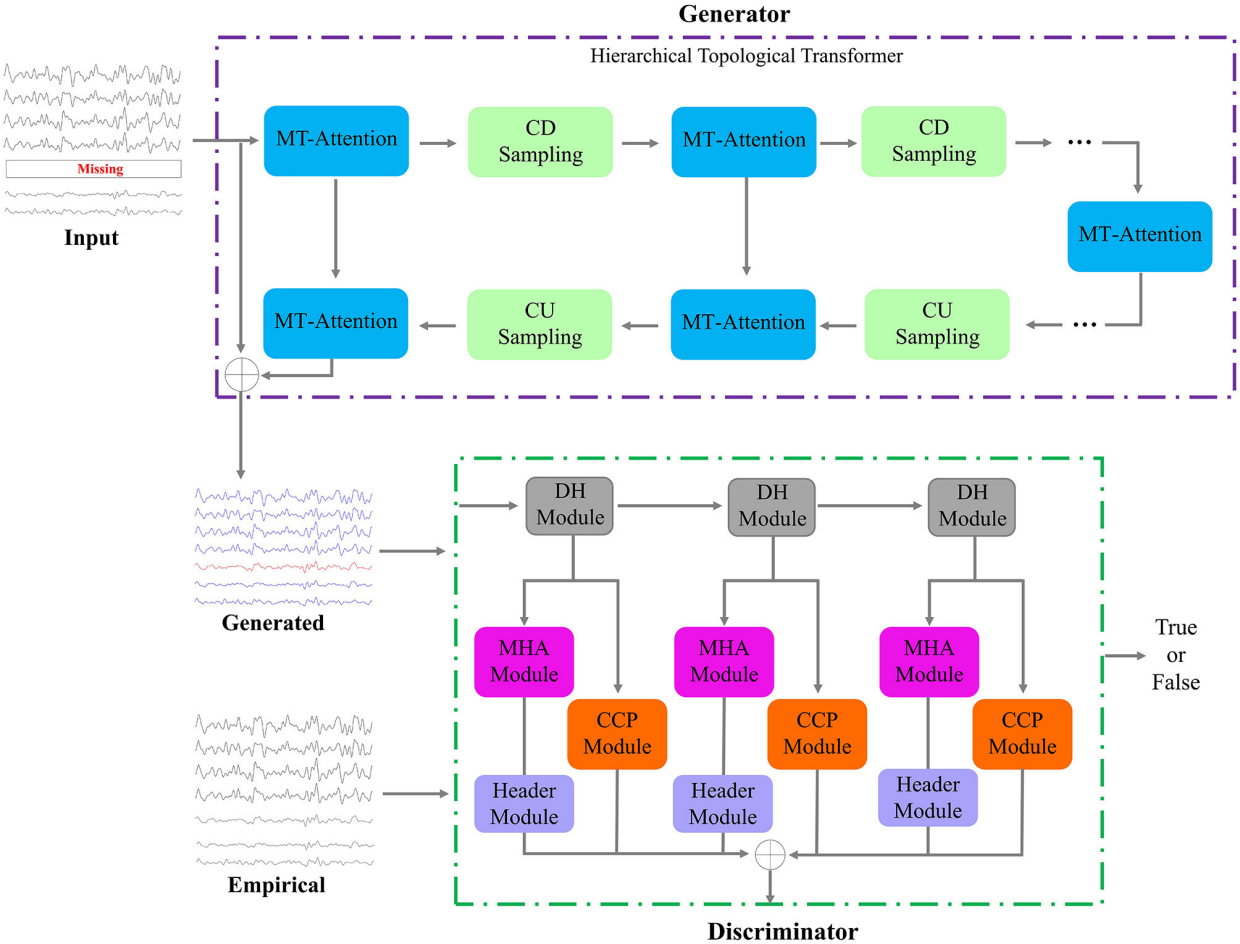
Framework of the proposed model. It consists of one generator and one discriminator. The input is a multi-ROI time-series with missing time-series, and the output of the generator is the reconstructed multi-ROI time-series. The discriminator distinguishes whether the multi-ROI time-series is generated or empirical.

The main framework of this study is shown in Figure 2. The proposed UCT-GAN model consists of a hierarchical topological transformer generator and a multi-resolution relational discriminator. Given fMRI with missing signals on some brain area, after preprocessing, we can obtain the input data of the proposed model. We denoted it as the incomplete multi-ROI time-series signal ${\text{S}}_{m}\in {\mathbb{R}}^{N\times T}$, where *N* is the ROI number and *T* is the scanning functional signal length. The transformer-based generator aims to extract hierarchical features to recover the missing ROI-based signal. The multi-resolution discriminator is utilized to constrain the generated time-series (${\text{S}}_{g}\in {\mathbb{R}}^{N\times T}$) as close as the impirical time-series (${\text{S}}_{e}\in {\mathbb{R}}^{N\times T}$). To ensure the generation's good performance, we design three loss functions to optimize the model's parameters, including the generative loss, the discriminative loss, and the multi-resolution consistency loss.

#### 3.2.1 Hierarchical topological transformer generator

The generator is a neural network architecture that combines the principles of hierarchical attention mechanisms from transformers with one-dimensional convolutional layers. This architecture can capture both global and local temporal information at different scales and is often used for processing sequences or time series data efficiently and effectively. In the generator, we designed multiple layers of multi-level temporal-correlated attention (MT-Attention) and convolutional sampling to explore hierarchical temporal features. The output is the generated multi-ROI time-series **S**_*g*_. Setting *L* convolutional down sampling (CDS) layers, there are also *L* layers of convolutional up sampling (CUS) and 2*L*+1 layers of MT-Attention. The computation process can be expressed by the following formula:


$$\begin{array}{l} \begin{array}{c} {\text{S}}_{g}=\text{G}\left({\text{S}}_{m}\right)=f\left(MTA\left({\text{S}}_{m}\right),CDS\left({\text{S}}_{m}\right),CUS\left({\text{S}}_{m}\right)\right) \end{array} \end{array}$$

Here, the symbol *f* means the calculation processes in the generator.

**Figure 3:**
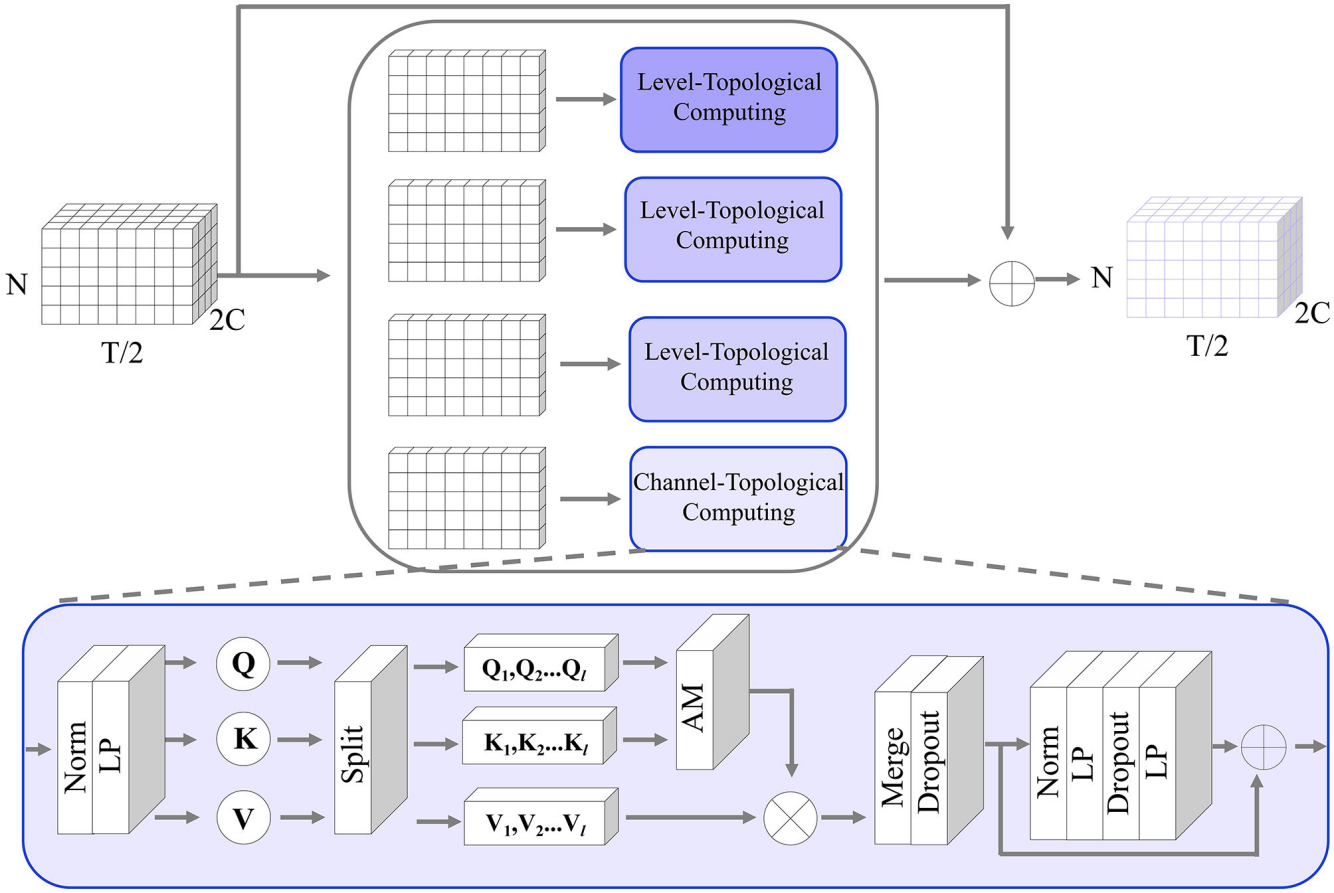
The detailed structure of the MT-Attention module in the generator. The input is a multi-channel ROI feature; by splitting along the channel direction, the channel topological computing pays attention to the temporal relationship between any pair of ROIs to recover the missing time-series. The output is the same size as the input.

Multi-level temporal-correlated attention (MTA) is an attention mechanism designed to capture dependencies and patterns at multiple levels of the characteristics of temporal sequences. This attention mechanism is especially useful for modeling time-series relationships between different ROIs. As shown in Figure 3, assuming the input of MT-Attention is the multi-ROI temporal feature **F**^*i*^ with the size 2*C*×*N*×*T*/2. We first split it into 2*C* slices, where each slice is sent to the level-topological computing (LTC) network to learn temporal dependencies between ROIs. For instance, some slices may represent lower levels and can be used to capture short-term dependencies within the sequence, while other slices may indicate higher levels and can be used to capture long-term dependencies. This multi-level structure allows MTA to consider different levels of temporal dynamics when reconstructing missing signals. Each slice is passed through the norm layer, linear projection (LP), splitting, attention map (AM), merge, dropout, norm, LP, and dropout. The output is the updated multi-ROI temporal feature **F**^*i*+1^ with the size of **F**^*i*^. The whole computation can be determined by


$$\begin{array}{l} \begin{array}{c} {\text{F}}^{i+1}=MTA\left({\text{F}}^{i}\right)=LTC\left({\text{F}}_{1}^{i}\right)||LTC\left({\text{F}}_{2}^{i}\right)||...||LTC\left({\text{F}}_{2C}^{i}\right) \end{array} \end{array}$$

where **F**^*i*^ means the input of the *i*-th module in the generator. || indicates the concatenation operation. **F**^*i*+1^ is the output of the *i*-th module in the generator. After seperating the 2C channels of **F**^*i*^, each channel component is represented as ${\text{F}}_{j}^{i}$. Here, *j* is in the range of 1 − 2*C*. These components are computed by the attention network and feedforward transform (FFT). The formula can be expressed as follows:


$$\begin{array}{l} \begin{array}{c} LTC\left({\text{F}}_{j}^{i}\right)=Attention\left({\text{F}}_{j}^{i}\right)+FFT\left({\text{F}}_{j}^{i}\right),\;j=\left\{1,2,...,2C\right\} \end{array} \end{array}$$

In the attention network, the norm is applied to the temporal feature to stabilize the training process. The LP layer is used to learn temporal attention matrices ***Q***, ***K***, ***V***. We applied *l* heads to the attention matrices and computed an attention map for each head. The attentioned heads are then merged by a LP layer and a dropout layer. Here is the computing formula:


$$\begin{array}{r} Attention(F_{j}^{i})=Dropout(LP(Att(Q_{1},K_{1},V_{1})||Att(Q_{2},K_{2},V_{2})||\ldots ||\\ Att(Q_{l},K_{l},V_{l}))) \end{array}$$

$$\begin{array}{l} \begin{array}{c} Att\left(Q_{l},K_{l},V_{l}\right)=\mathrm{softmax}\left(\frac{Q_{l}{K_{l}}^{\prime }}{\sqrt{T/\left(2l\right)}}\right)V_{l} \end{array} \end{array}$$

$$\begin{array}{l} \begin{array}{c} Q=\left(Q_{1}||Q_{2}||...||Q_{l}\right) \end{array} \end{array}$$

$$\begin{array}{l} \begin{array}{c} K=\left(K_{1}||K_{2}||...||K_{l}\right) \end{array} \end{array}$$

$$\begin{array}{l} \begin{array}{c} V=\left(V_{1}||V_{2}||...||V_{l}\right) \end{array} \end{array}$$

$$Q=LP(Norm(F_{j}^{i})),K=LP(Norm(F_{j}^{i})),V=LP(Norm(F_{j}^{i}))$$

In the feedforward transform network, it consists of Norm, LP, and dropout layers. They are used to provide a non-linear transformation to the intermediate representation produced by the self-attention mechanism. The LP layer projects the input temporal features from a lower-dimensional space to a higher-dimensional space, introducing some non-linearity in the process. The dropout layer aims to make the mapping weights more sparse for robust learning. The second linear layer then projects the result back to the original dimension. The computation formula is defined as follows:


$$FFT(F_{j}^{i})=Attention(F_{j}^{i})+LP(Dropout(LP(Norm(Attention(F_{j}^{i})))))$$

Convolutional sampling is utilized to reduce or increase temporal dimensions, including convolutional down (CD) sampling and convolutional up (CU) sampling. In the generator, the CD sampling halfs the dimension along the temporal direction while doubles the channels. The CU sampling doubles the dimension along the temporal direction while halving the channels.

For the CD sampling, one-dimensional convolutional kernels are applied to the multi-ROI temporal features. 1D convolution is used to capture local patterns or features within the time series data. By moving the filter across the sequence, it can detect changes, peaks, valleys, and other patterns within the temporal features. To reduce the dimensions, we set the stride step 2 and the doubled channels. For example, the input incomplete multi-ROI time-series signal **S**_*m*_ is sent to the generator. We treated it as the multi-ROI temporal features **F**_1_ with the size *C* × *N* × *T* (*C* = 1). After passing the MT-Attention module, the output **F**_1_ has the same size. Then, going through the CD sampling, the output **F**_2_ changed the size to 2*C* × *N* × *T*/2. For the CU sampling, we adopted transposed convolution to increase temporal dimensions.

#### 3.2.2 Multi-resolution discriminator

The multi-resolution discriminator aims to distinguish between generated multi-ROI time-series and empirical multi-ROI time-series. The discriminator's feedback can help optimize the generator. When the discriminator can easily distinguish between a true and false sample, it provides feedback to the generator to improve its generation capabilities. The generator then adjusts its parameters to produce a sample that is more similar to the true one.

The structure of the discriminator consists of three dimension halfving (DH) modules, three multi-head attention (MHA) modules, three header modules, and three central connectivity perception (CCP) modules. The generated/empirical multi-ROI time-series are first passed through the DH modules. For each DH module, the time-series dimension is halved but the channels are unchanged, which is different from the CD sampling in the generator. Through the three DH modules, the input multi-ROI time-series (e.g., **S**_*g*_) are resampled into three samples:


$$R_{1}=DH(S_{g})$$

$$R_{2}=DH(R_{1})$$

$$R_{3}=DH(R_{2})$$

where **R**_*i*_, *i* = {1, 2, 3} represents the high frequency signals (with the size *N*×*T*/2), middle frequency signals (with the size *N*×*T*/4), and low frequency signals (with the size *N*×*T*/8), respectively.

The resampled sample is sent to two branches: MHA and CCP. The former is used to capture the temporal dynamics and learn to measure temporal consistency; the latter is used to compute the consistency of missing-signal ROI-related connections. Both of them can contribute to the consistency measurement between the generated and empirical samples. Combining them can make the generated samples more realistic than the empirical samples. The MHA is the same as the transformer network with *l* heads. The header module transmits the attentioned temporal features into one scalar (1 means true, 0 means false). The CCP module first transforms the resampled sample into a connectivity matrix and then selects the missing-signal ROI-related connections. A one-layer LP is used to transmit the connectivity features into one scalar. The detailed computing steps are defined as follows:


$$o_{1}=Header(MHA(R_{1}))+CCP(R_{1})$$

$$o_{2}=Header(MHA(R_{2}))+CCP(R_{2})$$

$$o_{3}=Header(MHA(R_{3}))+CCP(R_{3})$$

$$o_{m}=\frac{o_{1}+o_{2}+o_{3}}{6}$$

where *o*_*i*_, *i* = {1, 2, 3} is the scalar. *o*_*m*_ is the final output score. After the model converges, the value of *o*_*m*_ approaches 0.5.

### 3.3 Hybrid loss functions

The adversarial loss, also known as the discriminator loss or the GAN loss, is a key component of a generative adversarial network (GAN). It quantifies how well the discriminator can distinguish between real and generated data. The goal of the generator is to minimize this loss, while the discriminator aims to maximize it. The adversarial loss is typically defined as a binary cross-entropy loss. The formulation of the adversarial loss is as follows:


$${ℒ}_{D}=||D(S_{e})-1|{|}^{2}+||D(G(S_{m}))|{|}^{2}$$

$${ℒ}_{G}=||D(G(S_{m}))-1|{|}^{2}$$

In addition, to keep the generated time-series as precisely similar as the empirical time-series, we introduced the multi-resolution consistency loss *L*_*MRC*_. It contains the reconstruction loss, the cross-correlation loss, and the topological loss at different temporal resolutions. The reconstruction error is a metric used to quantify the local dissimilarity between empirical time-series and generated time-series, and the cross-correlation loss can measure the overall temporal patterns between generated and empirical time-series. The topological loss computes the connectivity difference between the generated and empirical time-series. Here, we use the temporal mean absolute error (TMAE) and mean cross-correlation coefficient (MCC) to compute the loss functions. The multi-resolution consistency loss is defined as follows:


$$\begin{array}{l} {ℒ}_{MRC}=\sum_{k=0}^{3}TMAE(DH^{k}(S_{g}),DH^{k}(S_{e}))\\ \;+\sum_{k=0}^{3}MCC(DH^{k}(S_{g}),DH^{k}(S_{e}))\\ \;+\sum_{k=0}^{3}TMAE(PCC(S_{g}),PCC(S_{e})) \end{array}$$

$$TMAE(S_{g},S_{e})=\frac{1}{NT}\sum_{i=1}^{N}\sum_{j=1}^{T}|S_{g,ij}-S_{e,ij}|$$

$$MCC(S_{g},S_{e})=\frac{1}{N}\sum_{i=1}^{N}\frac{\sum_{j=1}^{T}\left(S_{g,ij}-S_{\bar{g},i})(S_{e,ij}-S_{\bar{e},i}\right)}{\sqrt{\sum_{j=1}^{T}{\left(S_{g,ij}-S_{\bar{g},i}\right)}^{2}}\sqrt{\sum_{j=1}^{T}{\left(S_{e,ij}-S_{\bar{e},i}\right)}^{2}}}$$

where the *DH*^*k*^ means stacking *k* DH layers. $S_{\bar{g},i}$ means averaging the *i*-th ROI time-series for **S**_*g*_. In summary, the total loss of the proposed UCT-GAN can be optimized by the following loss functions:


$${ℒ}_{all}={ℒ}_{G}+{ℒ}_{D}+\alpha {ℒ}_{MRC}$$

The detailed training pseudo-code is shown in Algorithm 1.

**Algorithm 1:**
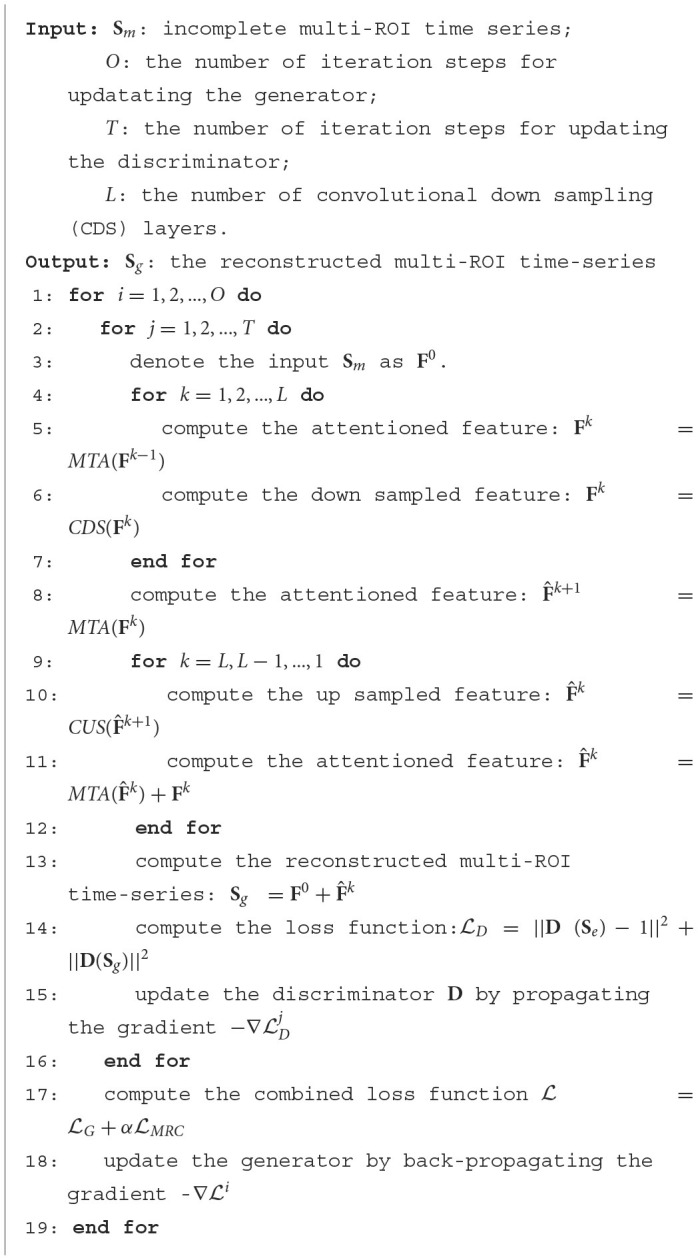
Optimizing the UCT-GAN model.

## 4 Experiments and results

### 4.1 Model settings and evaluating metrics

The UCT-GAN model is trained on Windows 11 using the pytorch deep learning framework to reconstruct the incomplete multi-ROI time-series. The parameter *L* is studied in the range of 1–10 to find the optimal value. In addition, the hyperparameter α in the loss functions is investigated to determine the best weighting of the multi-resolution consistency loss. During the training, we first train the discriminator and then train the generator. The learning rate for the generator and the discriminator is set at 3.*e*−4 and 1.*e*−4, respectively. The Adam was used to train the models with a batch size of 16. Overall, 10-fold cross verification is adopted to evaluate our model's reconstruction performance.

Measuring the similarity between generated and empirical time-series data is a crucial step in evaluating the performance of the proposed model. Three metrics can be used for this purpose, including mean absolute error (MAE), root mean square error (RMSE), coefficient of determination of the prediction (R2) (Ma et al., 2021), and dynamic time warping (DTW) (Philips et al., 2022). MAE measures the average absolute difference between the values of the generated and empirical time-series. It is calculated by taking the absolute difference between each corresponding pair of points in the generated and empirical time-series, summing these differences, and then dividing by the total number of data points. MAE is sensitive to outliers and provides a straightforward measure of the magnitude of errors. The formula is defined as follows:


$$\begin{array}{l} MAE=\frac{1}{N_{m}}\overset{N_{m}}{\underset{i=1}{\sum }}\overset{T}{\underset{j=1}{\sum }}|s_{\text{generated}}\left(ij\right)-s_{\text{empirical}}\left(ij\right)| \end{array}$$

RMSE calculates the square root of the average of the squared differences between the generated and empirical time-series. It provides a measure of the magnitude of errors and gives higher weight to larger errors because of the squaring operation. The formula is defined as follows:


$$\begin{array}{l} RMSE=\frac{1}{N_{m}}\overset{N_{m}}{\underset{i=1}{\sum }}\sqrt{\frac{1}{T}\overset{T}{\underset{j=1}{\sum }}{\left(s_{\text{generated}}\left(ij\right),s_{\text{empirical}}\left(ij\right)\right)}^{2}} \end{array}$$

where the *N*_*m*_ means the number of missing time-series ROIs. *s*_generated_(*ij*) is the *i*-th and *j*-th element in the **S**_*g*_, and the *s*_empirical_(*ij*) is the *i*-th and *j*-th element in the **S**_*e*_. The R2 measures how the reconstructed time-series linearly regresses the empirical time-series, and large value indicates good reconstruction performance. The DTW measures the distance between reconstructed and empirical time-series, where small values indicate good reconstruction performance.

### 4.2 Parameter analysis

**Figure 4:**
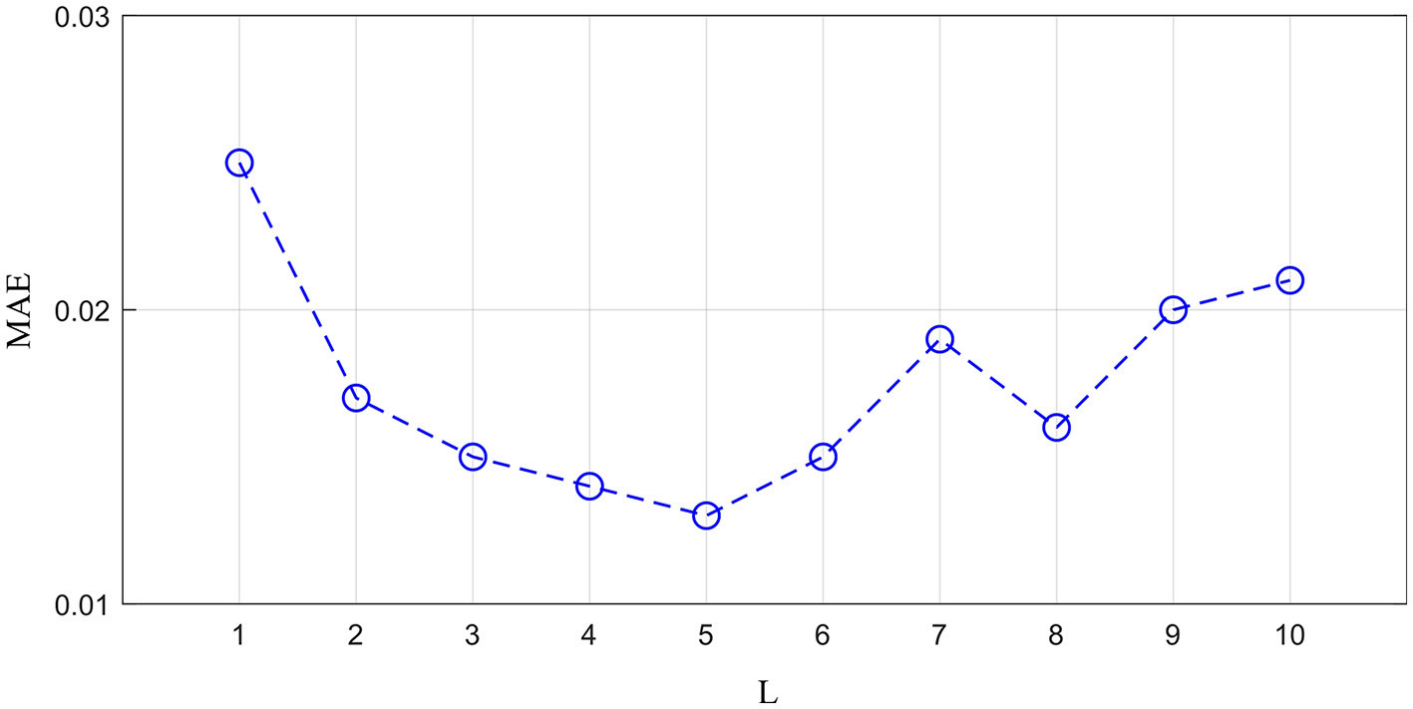
The impact of layer number *L* on the generator.

The generator is important for reconstructing missing ROI time-series. To explore the optimal MT-Attention layer number, we studied 10 values of *L* to determine the best value. We treated the left amygdala as the missing time-series ROI. The MAE is calculated by measuring the difference between the reconstructed time-series and the empirical time-series. As shown in Figure 4, the MAE changes as the *L* increases. The best value of *L* is 5. The smaller value of *L* with a large MAE may be the result of model underfitting, while the larger value of *L* with a large MAE may be the result of overfitting.

**Figure 5:**
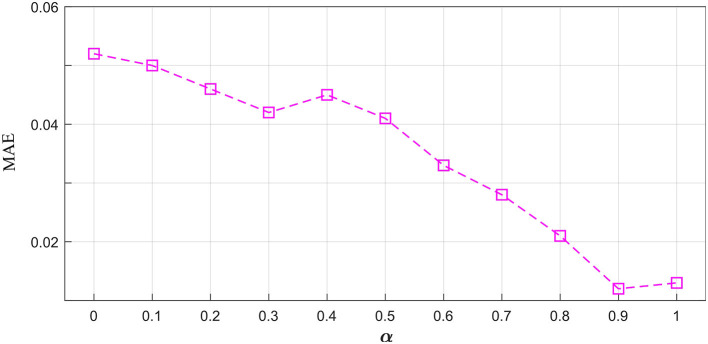
The influence of parameter α on the hybrid loss functions.

The proposed multi-resolution consistency loss can guarantee the model's good reconstruction performance. We investigated the optimal imporantce of *L*_*MRC*_ in the hybrid loss functions. As shown in Figure 5, we chose the value of α from 0.0 to 1.0. The 0.0 means the *L*_*MRC*_ is removed from the total loss. As the value of α increases, the MAE shows a downward trend. This indicates the importance of the proposed multi-resolution consistency loss in reconstructing the missing signals. The best value of α is achieved at 0.9. In the downstream tasks, we will evaluate the model's performance using the optimal *L* and α.

### 4.3 Reconstruction performance

**Figure 6:**
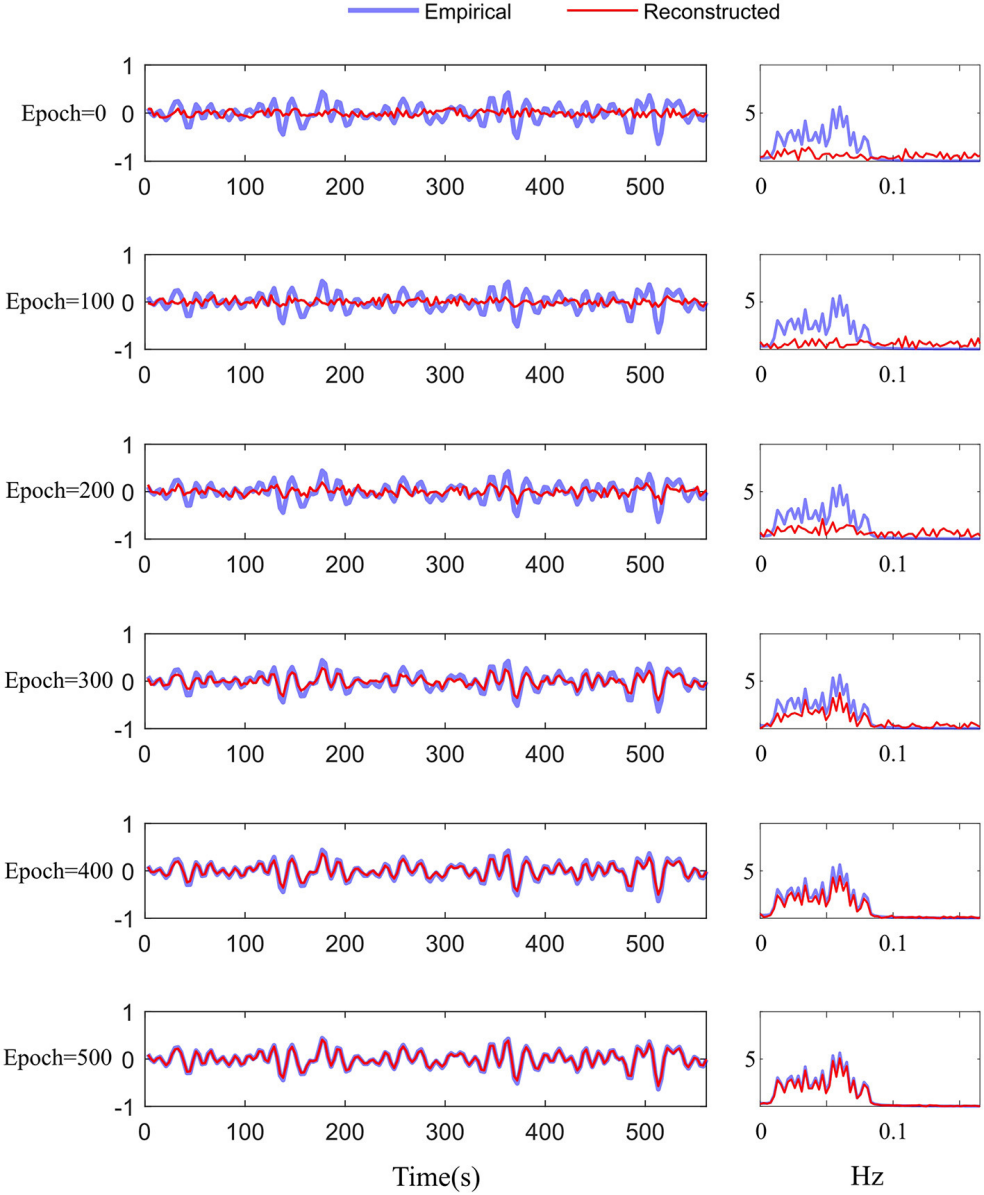
Training details of the difference between the empirical and reconstructed time-series in the time and frequency domains from 0 to 500 epochs.

**Figure 7:**
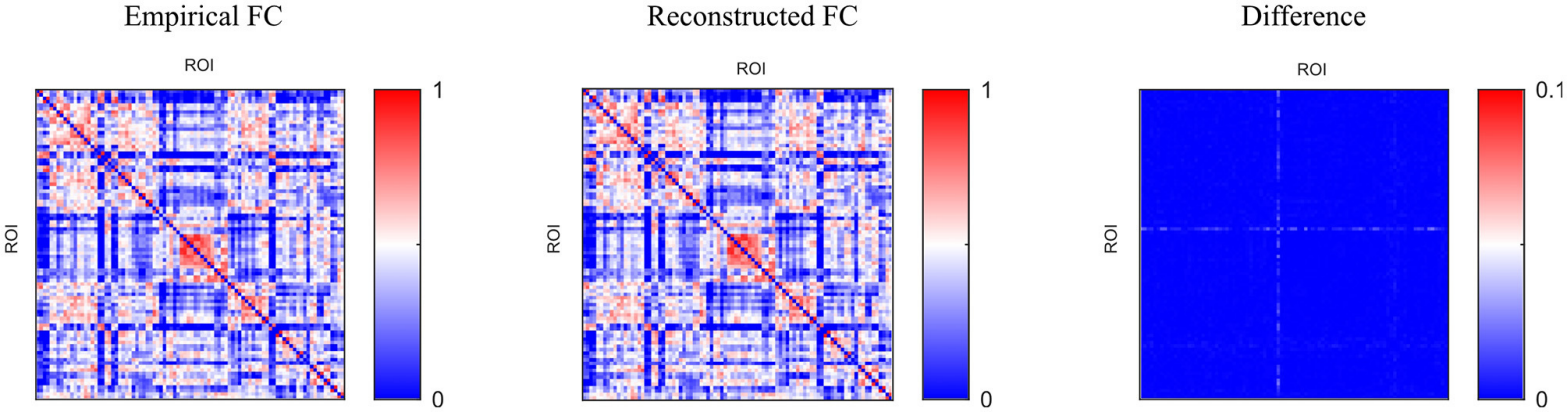
Comparison of functional connectivity using different methods.

We adopted the above settings and continued to investigate the time-series reconstruction performance of the left amygdala. We presented the training details about the reconstructing processes. As shown in Figure 6, we initialized the missing signal as Gaussian noise at the 0 epoch, and then the Gaussian noise is getting closer to the empirical signal as the epoch arrives at 500. The right column shows the frequency spectrum of the left column. The frequency spectrum is computed using the Fast Fourier transform, which converts the left time-series into individual spectral components and thereby provides frequency information about the it. At 0 epoch, the frequency information between empirical and reconstructed time-series is very different, and as the epoch increases, the frequency information difference gradually decreases; at the final epoch, the frequency information between the two signals is almost the same, indicating good reconstruction result. Furthermore, we quantitatively evaluate the functional connectivity using the PCC computed by empirical and generated time series. Figure 7 shows that the larger difference is the missing-signal ROI-related connections in the right column. The maximum PCC change is lower than 0.05, which has little influence on brain network analysis.

**Figure 8:**
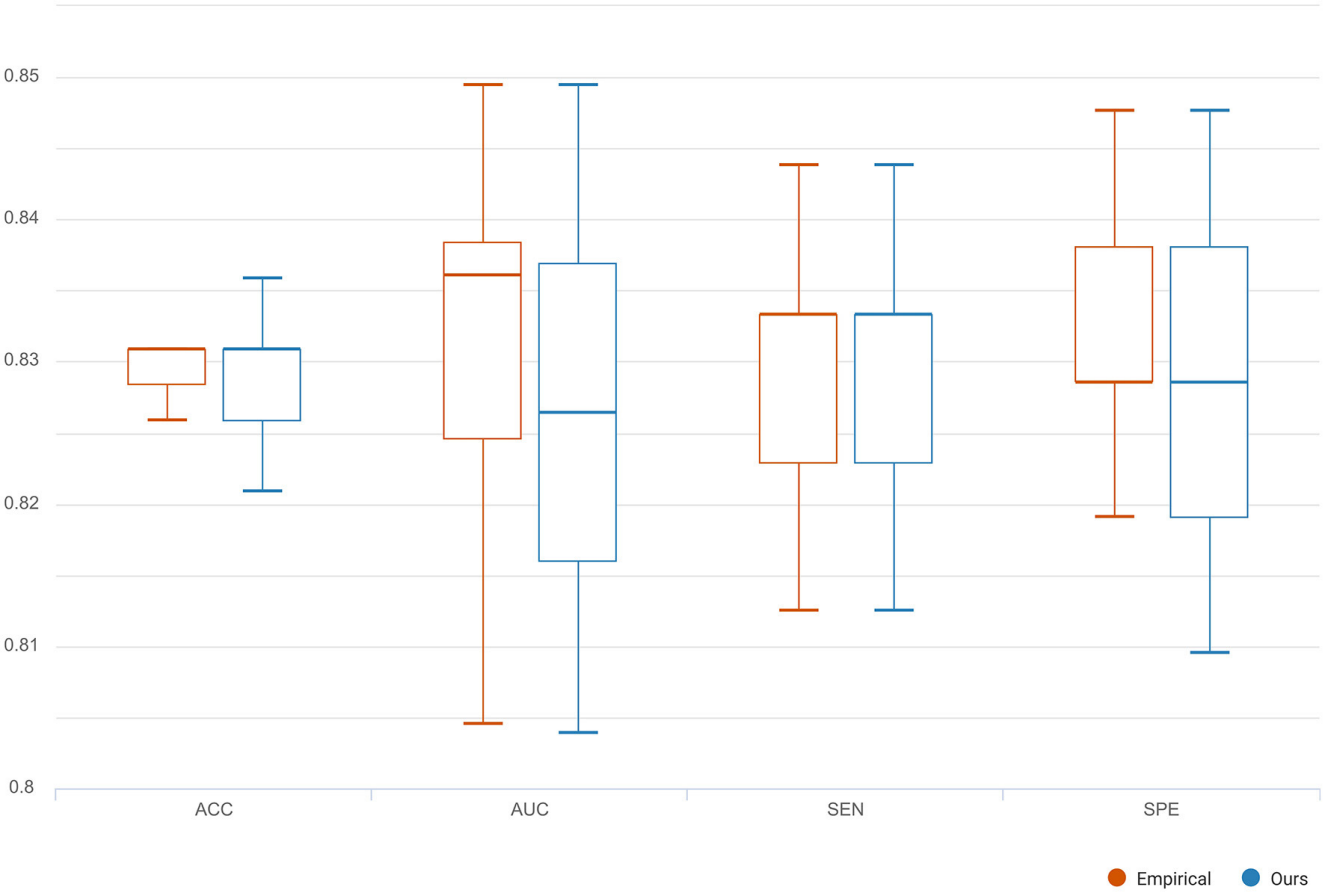
The classification comparison of functional connectivity constructed by empirical and reconstructed time-series, respectively.

To compare the reconstruction performance using different models, we chose the six competing models: (1) C-RNN-GAN (Mogren, 2016), (2) RCGAN (Esteban et al., 2017), (3) waveGAN (Donahue et al., 2018), (4) TimeGAN (Yoon et al., 2019), (5) SigCWGAN (Ni et al., 2020), and (6) TCGAN (Xia et al., 2023). The input is the incomplete multi-ROI time-series with only one ROI time-series removed. We compared the reconstructed missing signal by computing the three metrics: MAE, RMSE, R2, and DTW. We have randomly split the dataset into 10 folds 10 times. For each method, we calculate the mean and standard deviation for the four metrics. As shown in Table 1, the GAN-based models show inferior performance than the transformer-based models. The possible reason is that the transformer benefits from the relationship modeling ability. Among these methods, the proposed model combining the transformer and GAN achieves the best reconstruction performance in terms of MAE (0.010), RMSE (0.015), R2(0.998), and DTW (1.872). To prove the effectiveness of the reconstructed time-series, we constructed functional connectivity (FC) from empirical and reconstructed time-series, respectively. The constructed FC was then sent to the BrainNetCNN (Kawahara et al., 2017) classifier to compute four metrics (i.e., ACC, SEN, SPE, and AUC) for NC versus LMCI. The classification results are shown in Figure 8, and there is no significant difference between the four metrics. The p-values of ACC, SEN, SPE, and AUC between the two methods are 0.894, 0.756, 0.703, and 0.358, respectively. The p-values are larger than 0.05, indicating that the proposed method can achieve the same classification performance as the empirical method. Furthermore, we investigated different methods' robustness to the noise, and we added Gaussian noise (1 or 5%) to the training and testing data. Then, we calculated the mean and standard deviation values for the seven methods. As shown in Table 2, our model achieves the best classification performance among the seven methods with a small deviation error, indicating our model's effectiveness and robustness.

**Table 1:** The reconstruction performance using different models.

**Method**	**MAE ↓**	**RMSE ↓**	**R2 ↑**	**DTW ↓**
C-RNN-GAN	0.030 ± 0.017	0.036 ± 0.026	0.960 ± 0.014	5.642 ± 0.513
RCGAN	0.026 ± 0.015	0.033 ± 0.023	0.966 ± 0.010	4.835 ± 0.449
waveGAN	0.022 ± 0.014	0.029 ± 0.021	0.971 ± 0.008	3.578 ± 0.416
TimeGAN	0.016 ± 0.012	0.023 ± 0.018	0.987 ± 0.006	3.043 ± 0.372
SigCWGAN	0.014 ± 0.011	0.020 ± 0.015	0.989 ± 0.005	2.644 ± 0.301
TCGAN	0.013 ± 0.010	0.019 ± 0.014	0.992 ± 0.003	2.302 ± 0.221
Proposed	**0.010** ±**0.006**	**0.015** ±**0.011**	**0.998** ±**0.001**	**1.864** ±**0.247**

The best results are in bold.

**Table 2:** The reconstruction performance using different models for different noise levels.

**Noise level**	**Method**	**MAE ↓**	**RMSE ↓**	**R2 ↑**	**DTW ↓**
1%	C-RNN-GAN	0.032 ± 0.018	0.038 ± 0.027	0.951 ± 0.014	5.813 ± 0.503
	RCGAN	0.029 ± 0.014	0.036 ± 0.022	0.954 ± 0.011	4.911 ± 0.432
	waveGAN	0.023 ± 0.013	0.030 ± 0.022	0.970 ± 0.009	3.415 ± 0.413
	TimeGAN	0.017 ± 0.012	0.025 ± 0.019	0.976 ± 0.007	3.112 ± 0.367
	SigCWGAN	0.015 ± 0.012	0.021 ± 0.016	0.978 ± 0.006	2.697 ± 0.304
	TCGAN	0.013 ± 0.011	0.019 ± 0.016	0.991 ± 0.005	2.303 ± 0.231
	Proposed	**0.010** ±**0.007**	**0.015** ±**0.012**	**0.998** ±**0.001**	**1.867** ±**0.249**
5%	C-RNN-GAN	0.037 ± 0.021	0.041 ± 0.029	0.941 ± 0.028	6.219 ± 0.627
	RCGAN	0.032 ± 0.019	0.039 ± 0.027	0.948 ± 0.014	5.237 ± 0.507
	waveGAN	0.027 ± 0.016	0.033 ± 0.025	0.964 ± 0.010	3.772 ± 0.486
	TimeGAN	0.019 ± 0.014	0.028 ± 0.021	0.971 ± 0.008	3.348 ± 0.371
	SigCWGAN	0.017 ± 0.013	0.024 ± 0.018	0.970 ± 0.008	2.713 ± 0.319
	TCGAN	0.015 ± 0.012	0.021 ± 0.017	0.985 ± 0.007	2.516 ± 0.229
	Proposed	**0.011** ±**0.006**	**0.016** ±**0.012**	**0.996** ±**0.002**	**1.896** ±**0.252**

The best results are in bold.

**Figure 9:**
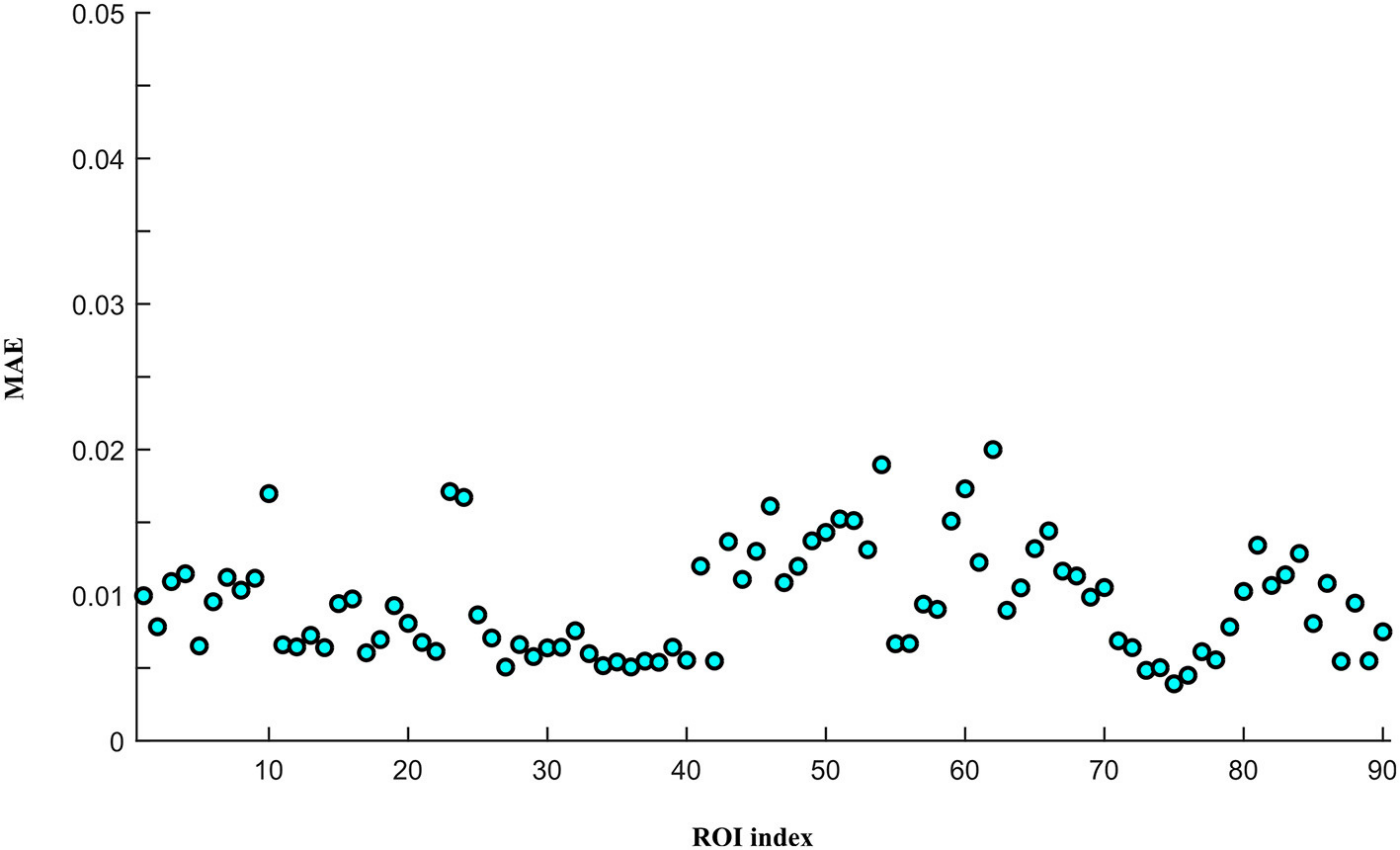
The reconstruction performance of different ROIs.

**Figure 10:**
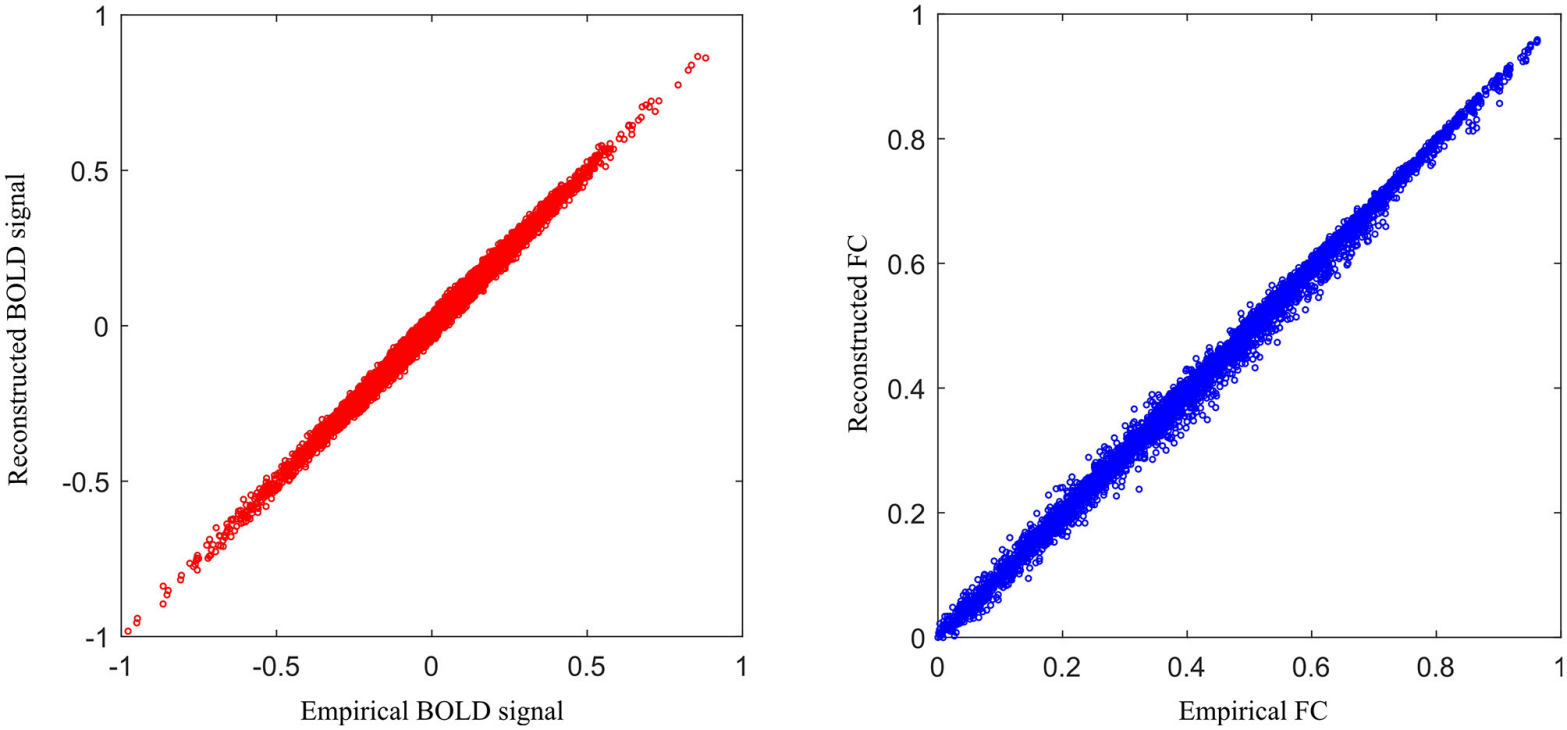
The relationship between empirical and generated results for all the ROIs.

To assess the reconstruction performance of other ROIs, we iteratively removed one ROI time-series from the preprocessed empirical multi-ROI time-series and trained them with our model. The reconstructed ROI time-series is compared with the empirical ROI time-series by computing the MAE value. The results are shown in Figure 9. For each ROI, we displayed the MAE. The mean MAE value of all ROIs is 0.010, with an averaged standard error of 0.003. A large MAE of ROI may indicate that this ROI has high degrees. The removal of this ROI fails to model long-range relationships in the generator. To display all the ROI reconstruction performance, Figure 10 shows the correlation between empirical and generated time-series. The left sub-figure displays the correlation between empirical and generated time-series. We remove one ROI time-series and reconstruct it using other ROIs' time-series. For the convenience of display, we utilize the time-series of all ROIs from one subject to plot correlation between reconstructed and empirical BOLD signal. The right sub-figure shows the correlation between empirical and removed ROI-related connections. After reconstructing the removed ROI time-series, we compute the Pearson correlation coefficient between the removed ROI and other ROIs. All the computed connections from the corresponding subject are denoted as reconstructed functional connectivity (FC), which is compared with empirical FC. The two subfigures demonstrate our model's reconstruction ability. The reconstructed signals can be applied to the downstream brain network analysis.

### 4.4 Ablation study

To investigate the influence of the generator and the loss function on the reconstruction performance, we design four variants of the proposed model. (1) UCT by removing the discriminator from the UCT-GAN model. (2) UCT-GAN without hierarchical topological transformer (MSETD w/o HTT). In the generator, we removed the CD sampling and CU sampling and only kept one MT-attention block. (3) UCT-GAN without the multi-resolution consistency loss (MSETD w/o MRC). In the discriminator, we removed two DH modules and (4) the proposed UCT-GAN model. For each variant, we compute the mean value of MAE, RMSE, R2, and DTW. The results are shown in Table 3. Removing the hierarchical structure or the discriminator greatly reduces the time-series reconstruction performance, which shows the effectiveness and necessity of the proposed model in time-series restoration. The multi-resolution consistency also lowers the model's reconstruction performance to some extent. All of them contribute a lot to time-series reconstruction performance. It indicates that the U-shaped generative architecture and multi-resolution consistency loss capture the spatial and temporal characteristics, thus effectively restoring complex brain functional dynamics.

**Table 3:** Influence of different model's module on the reconstruction performance.

**Method**	**MAE**	**RMSE**	**R2**	**DTW**
UCT	0.025	0.031	0.952	3.981
UCT-GAN w/o HTT	0.021	0.028	0.968	3.415
UCT-GAN w/o MRC	0.018	0.025	0.974	3.153
**UCT-GAN**	**0.010**	**0.015**	**0.998**	**1.864**

The best results are in bold.

## 5 Discussion

**Figure 11:**
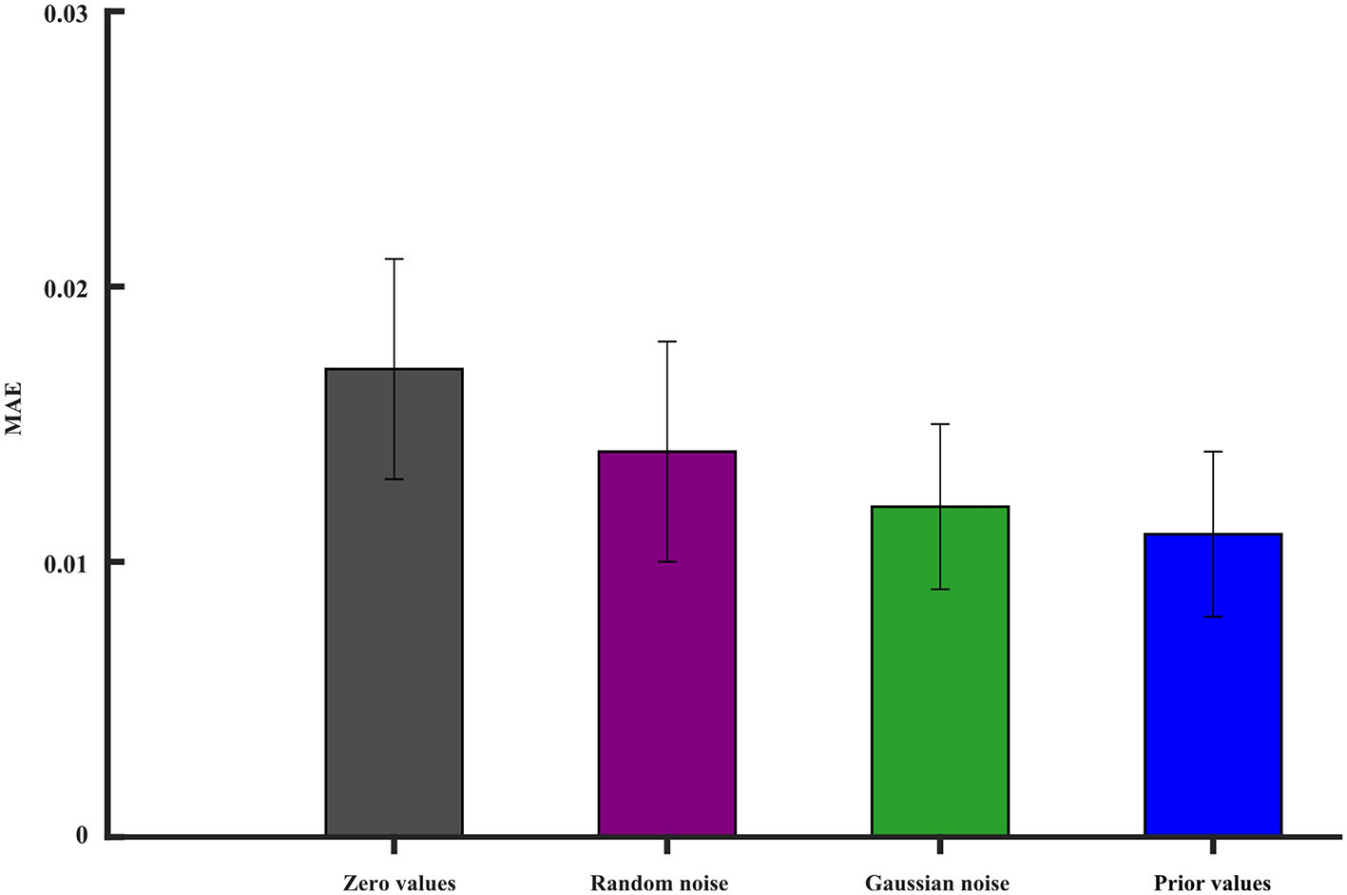
The impact of different initial values on the model reconstruction performance. The vertical line segment represents the margin of error.

In deep learning, neural networks are often non-convex and have multiple local minima. The choice of initial values can influence whether the optimization algorithm gets stuck in a poor local minimum or finds a more optimal solution. We investigate the iterative initial values during the training. As we know, the proper initial iterative values tend to find the optimal solution of the model. There are many strategies that are used to initialize the model's parameters' weights. We still study the condition when one ROI signal is missing. The missing signal is replaced by (1) zero values, (2) random noise, (3) Gaussian noise, and (4) prior values, which represent the averaged values of other ROI time series. All the initial values are forced into the range of 0 − 1. The MAE is used to evaluate the reconstruction performance. Figure 11 gives the best initial strategy of using the prior values. The prior value strategy can mitigate the risk of convergence to suboptimal solutions.

**Figure 12:**
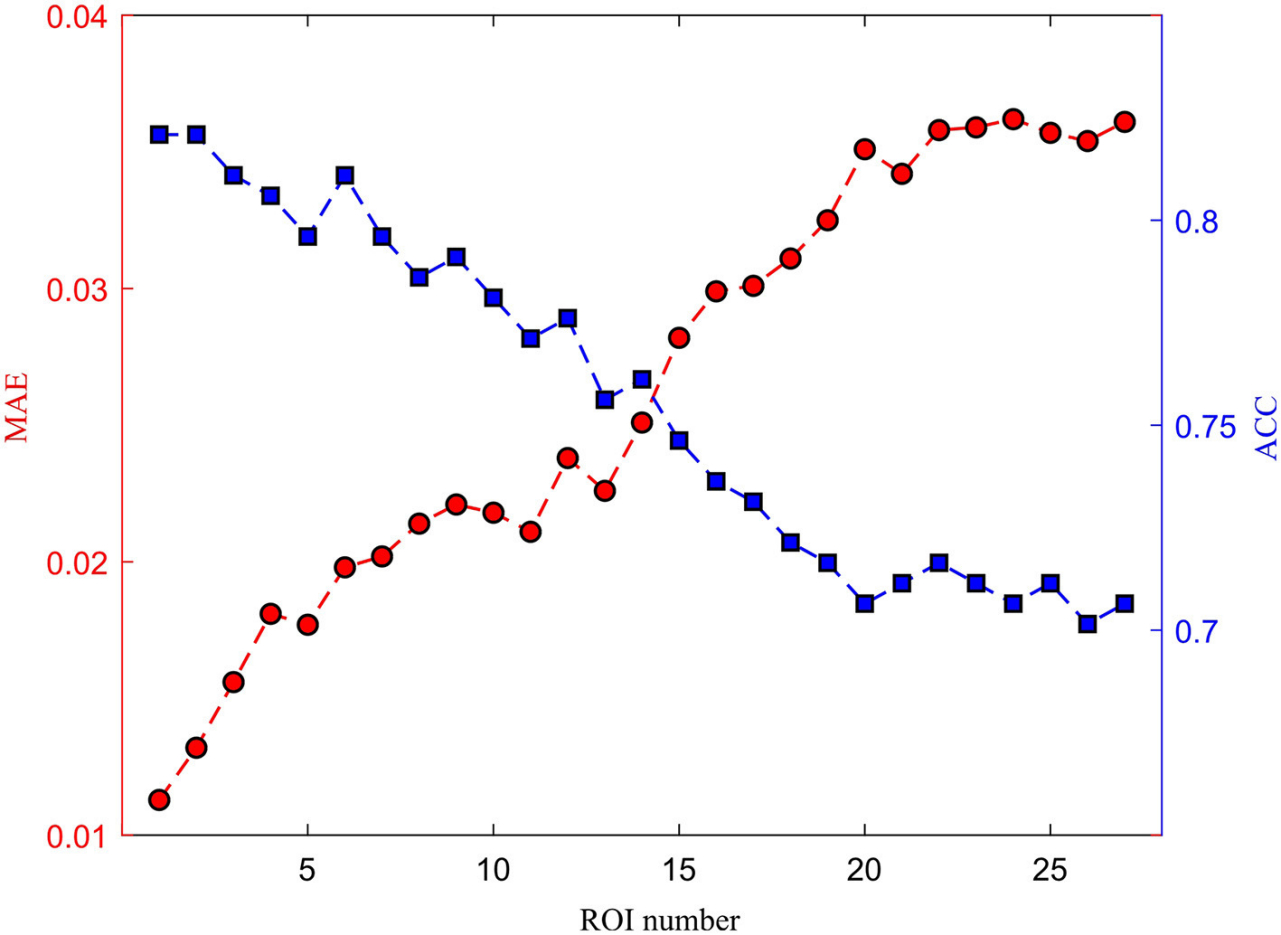
The effect of different damaged ROIs on the reconstruction performance.

The medical treatment using the DBS usually involves implanting a device into the brain to alleviate symptoms of various neurological disorders. The intersection of the fornix and stria terminalis in the brain may be the optimal area for DBS treatment. The stria terminalis serves as a major output pathway of the amygdala. Therefore, we investigated the amygdala for potential clinical applications. The damaged signals in the amygdala may also influence the adjacent ROIs, such as the up, down, left, and right brain areas. We cumulatively removed one ROI signal from the scanned fMRI and evaluated the reconstruction performance. As shown in Figure 12, as the number of missing brain regions increases, the MAE gradually increases and the ACC correspondingly decreases. This shows that the reconstruction ability is greatly reduced.

The proposed model combines the U-shaped convolutional transformer and GANs to restore the missing brain functional time series. By restoring complex brain functional dynamics, the proposed model can achieve the same classification results as the empirical method. More missing signals can greatly reduce the reconstruction performance and disease prediction. No more than two ROI missing signals probably have little influence on the dementia diagnosis and brain network analysis. Though the proposed model can achieve good restoration performance, there are two limitations. One limitation is that the studied ROI may have a larger volume than that of the real distortion brain region. In the future, we will try more fined atlas to investigate the BOLD signal distortion, since more fined ROIs can better describe the signal distortion and help precisely reconstruct the missing signals for improving disease analysis. Another one is that the proposed model is tested theoretically with small subjects. In the next study, we will validate our model on a larger dataset, such as the UK Biobank dataset (https://www.ukbiobank.ac.uk/).

## 6 Conclusion

This study proposes a novel U-shaped convolutional transformer GAN (UCT-GAN) model to restore the missing brain functional time-series data. By leveraging generative adversarial networks (GANs) and the U-shaped transformer architecture, the proposed UCT-GAN can effectively capture hierarchical features in the restoration process. It should be stressed that the multi-level temporal-correlated attention and the convolutional sampling in the generator capture the long-range and local temporal features of the missing signal and associate their relationship with the effective signal. We also designed a multi-resolution consistency loss to learn diverse temporal patterns and maintain consistency across different temporal resolutions. We theoretically tested our model on the public Alzheimer's Disease Neuroimaging Initiative (ADNI) dataset, and our experiments demonstrate superior reconstruction performance with other competing methods in terms of quantitative metrics. The proposed model offers a new solution for restoring brain functional time-series data, driving forward the field of neuroscience research through the provision of enhanced tools for data analysis and interpretation.

## Data availability statement

Publicly available datasets were analyzed in this study. This data can be found at: https://adni.loni.usc.edu.

## Author contributions

QZ: Conceptualization, Data curation, Funding acquisition, Methodology, Writing – original draft, Writing – review & editing. RL: Data curation, Software, Visualization, Writing – review & editing. BS: Data curation, Software, Visualization, Writing – review & editing. JH: Conceptualization, Methodology, Software, Writing – review & editing. YZ: Investigation, Resources, Validation, Writing – review & editing. XC: Formal analysis, Resources, Validation, Writing – review & editing. YW: Formal analysis, Investigation, Writing – review & editing. JG: Funding acquisition, Project administration, Supervision, Writing – review & editing.

## Footnotes

^1^ http://adni.loni.usc.edu/
